# A Comparative Study on Outcome of Government and Co-Operative Community-Based Health Insurance in Nepal

**DOI:** 10.3389/fpubh.2017.00250

**Published:** 2017-09-22

**Authors:** Chhabi Lal Ranabhat, Chun-Bae Kim, Dipendra Raman Singh, Myung Bae Park

**Affiliations:** ^1^Department of Preventive Medicine, Yonsei University, Wonju College of Medicine, Wonju, South Korea; ^2^Institute for Poverty Alleviation and International Development, Yonsei University, Wonju, South Korea; ^3^Health Science Foundations and Study Centre, Kathmandu, Nepal; ^4^Ministry of Health, Public Health, Monitoring and Evaluation Division, Kathmandu, Nepal; ^5^Department of Gerontology, Health and Welfare, Pai Chai University, Daejeon, South Korea

**Keywords:** community-based health insurance, co-operative, benefit package, social inclusion, healthcare, Nepal

## Abstract

**Background:**

There are different models for community-based health insurance (CBHI), and in Nepal, among them, the government and the local communities (co-ops) are responsible for operating the CBHI models that are in practice.

**Aims:**

The aim of this study is to compare the outcomes in relation to benefit packages, population coverage, inclusiveness, healthcare utilization, and promptness of treatment for the two types of CBHI models in Nepal.

**Methods:**

This study was an observational and interactive descriptive study using the concurrent mixed approach of data collection, framing, and compilation. Quantitative data were collected from records, and qualitative data were collected from key informants in all 12 CBHI groups. Unstructured questionnaires, observation checklists, and memo notepads were used for data collection. Descriptive statistics and the Mann–Whitney *U* test were used when appropriate. Ethically, written informed consent was obtained from the respondents who participated in the study, and they were told that they could withdraw from the study anytime.

**Results:**

The study revealed the following: new enrolment did not increase in either group; however, the healthcare utilization rate did (Government 107% and co-ops 137%), while the benefit packages remained almost same for both groups. Overall, inclusiveness was higher for the government group. For the CBHI co-ops, enrollment among the religious minority and the discount negotiated with the hospitals for treatment were significantly higher, and the promptness in reaching a hospital was significantly faster (*p* < 0.05) than that in the government-operated CBHI.

**Conclusion:**

Findings indicate that CBHI through co-ops would be a better model because of its lower costs and ability to enhance self-responsiveness and the overall health system. Health insurance coverage is the most important component to achieve universal health coverage.

## Introduction

Community-based health insurance (CBHI) is attracting attention in low- and middle-income countries as a means for improving healthcare utilization and protecting households against impoverishment caused by out-of-pocket medical expenditures. The World Health Organization and the World Bank have continuously suggested reducing out-of-pocket payments (OPPs) and promoting universal health coverage (UHC) (1, 2). Different health financing approaches have been developed to counter the detrimental effects of user fees introduced in the 1980s, but those efforts have not yet increased healthcare utilization, particularly among marginalized populations and, moreover, sometimes lead to catastrophic health expenditures (CHEs) (2–4). There are different models of health insurance; among them, CBHI is the most widely used among middle-income populations and in remote areas. CBHI has been implemented on a small scale in Nepal, but its effectiveness remains a critical question.

There are different models of health insurance. Mandatory and single payer health insurance models are considered to be among the better approaches, but it is very difficult to collect premiums in low- and middle-income countries due to a profound dearth of economic activities in the informal sector and a lack of information technology that is compatible with premiums (5). Likewise, for people who are ultra-poor and live in rural areas, enrolling in health insurance is an additional challenge. The government-only approach is unable to provide universal health insurance because the primary responsibility for health falls upon each individual. In the United States, the Obama administration proposed reducing healthcare costs and providing affordable health service using a co-operative concept (6); and co-operative health insurance is also replacing private health insurance in Canada (7). Voluntary health insurance could be a step forward to lower risks associated with illness.

There are two approaches to voluntary health insurance that have been initiated by both the government and local communities. Some studies have investigated the outcomes of CBHI in low- and middle-income countries, but their results have been inconsistent. Several previous reviews have evaluated the performance of CBHI in terms of enrollment, financial management, and sustainability (8–10). One study from Laos indicated that the government-funded CBHI has low coverage, but the insured people have a significantly higher level of healthcare utilization, lower OPPs, lower incidence of catastrophic expenditures, and a lower propensity to employ coping mechanisms (11). In a study in Ethiopia, out-patient department (OPD) services increased, while inpatient department (IPD) services remained the same even after the implementation of CBHI (12). However, people were not convinced that they should enroll in CBHI, and the coverage rate was thereby unsatisfactory.

There are some successful reports of voluntary health insurance through community initiatives, such as in Vietnam (13) and Japan (14), and government initiatives in some developing countries. In China, a New Co-operative Medical Scheme (NCMS) was piloted, and it had mixed results (15). Similarly, the state government of Karnataka in India also made health insurance available to its people and tried to reduce OPPs and CHE (16). Previous studies have largely focused on the single model of CBHI, and comparison of outcomes between government and co-operative CBHI models is rare globally, and none has focused on Nepal.

Over the past two decades, the government of Nepal has spent less than 5% of the total budget on health care, while more than two thirds of the population used OPPs during illness (17) and over 13% had CHEs due to vehicular injuries, diabetes, asthma, and/or heart disease (18, 19). In Nepal, more than 80% people live in villages, and the livelihood of 75% of these people is subsistence agriculture; hence, they cannot purchase any medical care, and to date, health insurance has covered only less than 5% of total population (20). For example, in the case of child pneumonia, the direct cost for treatment per case was $25, and its indirect cost was $312, indicating that there is a great loss when there is illness in a family (21). Until now, more than one-fourth of the population has used endogenous and complementary and alternative therapy (22). Obviously, the government does not have sufficient funds to finance all health expenditure for the entire population.

Lack of sustainable health financing has resulted in highly unequal health care, and such inequalities affect both rich and poor, urban and rural, male and female, and upper caste and disadvantaged groups. In Nepal, there have been several experiments with CBHI reported since 2004 in both rural and urban settings (23). Provider-based health insurance was introduced in Nepal in 2003 through six pilot schemes offered by the government. At the same time, some community groups (co-ops) started CBHI on their own initiative supported by non-governmental organizations (NGOs) (24). CBHI schemes in Nepal complemented a number of specialized programs to improve access to healthcare services.

The aim of this study is to compare the outcomes of CBHIs in Nepal, both CBHIs offered by government health facilities and those offered by community groups, in terms of benefit packages, population coverage, inclusiveness, healthcare utilization, and promptness of treatment.

## Materials and Methods

### Study Design

This study was an observational and interactive descriptive study composed of qualitative and quantitative data sources.

### Methods of Study

After crystallizing the research outcomes, a study team was established. The study area, tools, respondents, data collection methods, data analysis, and synthesizing approach were finalized. Study team included all authors, statisticians, field supervisors, and community rapport builders. We used a combined descriptive research method, which consisted of quick observations and interactive methods with respondents for higher reliability and an applicable approach in which each part complemented the other in the operational research (25, 26).

### Study Approaches

**Figure 1:**
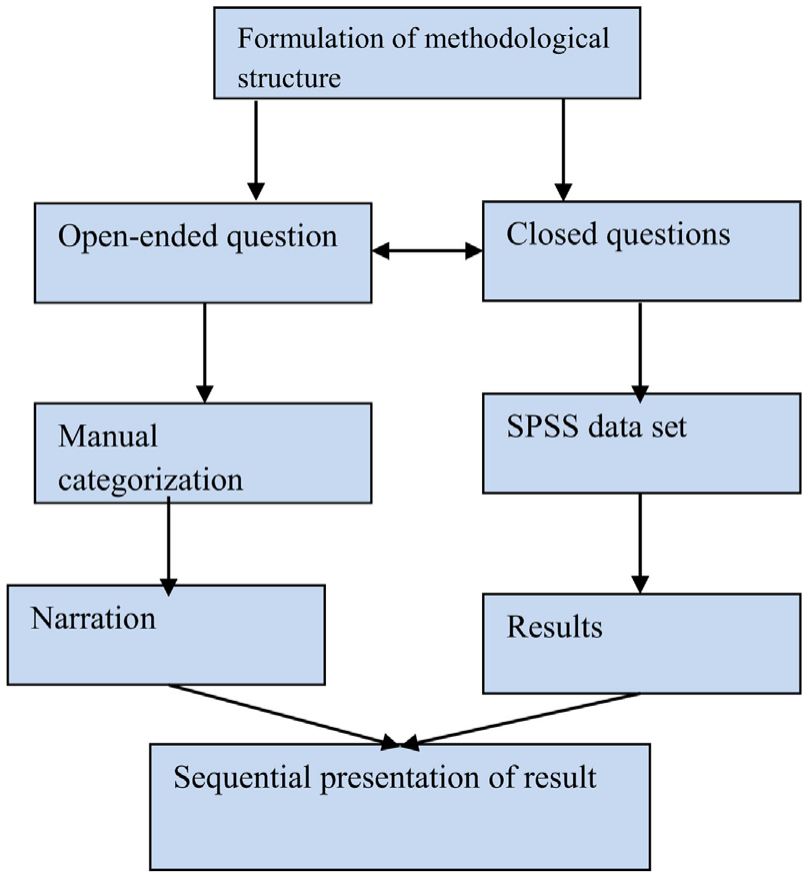
Concurrent mix study design: Driscoll (28).

A mixed method approach would be appropriate for both qualitative and quantitative data comparisons between two groups and for synthesizing the results (27). We used Driscoll et al.’s concurrent structure survey by applying open-ended and closed-ended questions with the same respondents (28) (Figure 1). This method integrates research questions, employs rigorous quantitative research assessing magnitude, and explores the meaning and understanding of concepts; it utilizes multiple processes, combines these to draw on their strengths, and frames the investigation within philosophical and theoretical positions (29). Recently, Creswell (30) emphasized that such an approach is better for philosophical, theoretical, and methodological perspectives. Qualitative research seeks to understand how individuals explore and perceive their experiences. Quantitative research is more powerful for generalization of the findings, whereas a combined approach is more valid, reliable, and replicable. Thus, the mixed impact of both designs is more powerful than a single approach.

### Study Sampling and Characteristics of Respondents

We used all (not selected) volunteer-based health insurance groups, both government-operated CBHI (6) and co-operative groups CBHI (6). Both types of CBHIs are demonstration projects regulated by the Ministry of Health (MoH), Nepal. Other characteristics are discussed below (Table 1).

**Table 1:** Characteristics of data and respondents.

Characteristics		Government community-based health insurance (CBHI)	Co-ops CBHI
Quantitative data approach		From the record registers	From the record registers

Qualitative data approach	No. of key informants	Two for each CBHI: chairperson and focal person (12)	Two for each CBHI; member secretary and chairperson (12)

	Key informant guidelines	Used and similar	Used and similar

	Recorder	Used	Used

Involvement of stakeholders		No	No

### Study Setting

Being a concurrent mixed study, we obtained data from institution and opinions from key informants. The institutions were selected purposively, and totally 12 institutions were included, of which 6 were government health centers and 6 were co-ops conducting CBHI. The qualitative information was collected from the responsible (focal) person in each government health center and the member secretary of co-ops and chairperson of health center and co-operative group. Their responses were categorized and presented in a narrative form after the data results and summary statements were presented by each group.

### Study Group and Population

We selected two models of CBHI in Nepal.

#### Government-Run, CBHI (Group A)

This group had six pilot areas, governed by the MoH of Nepal, based on a population survey done more than 5 years before. Primary health centers and district-level hospitals offered benefit packages of health insurance using their own management. They operated on government financing.

#### Co-Operative Prepayment Health Organization (Group B)

This group had six community groups governed by these communities in collaboration with private and government hospitals. This model can be defined as a zero-cost financing model that empowers these community groups. There was some support from NGOs, but this was only in-kind support. Funding came from their regular savings, subsidies, and donations from other organizations, and some amount of benefits came from group income generation activities.

### Study Area

**Figure 2:**
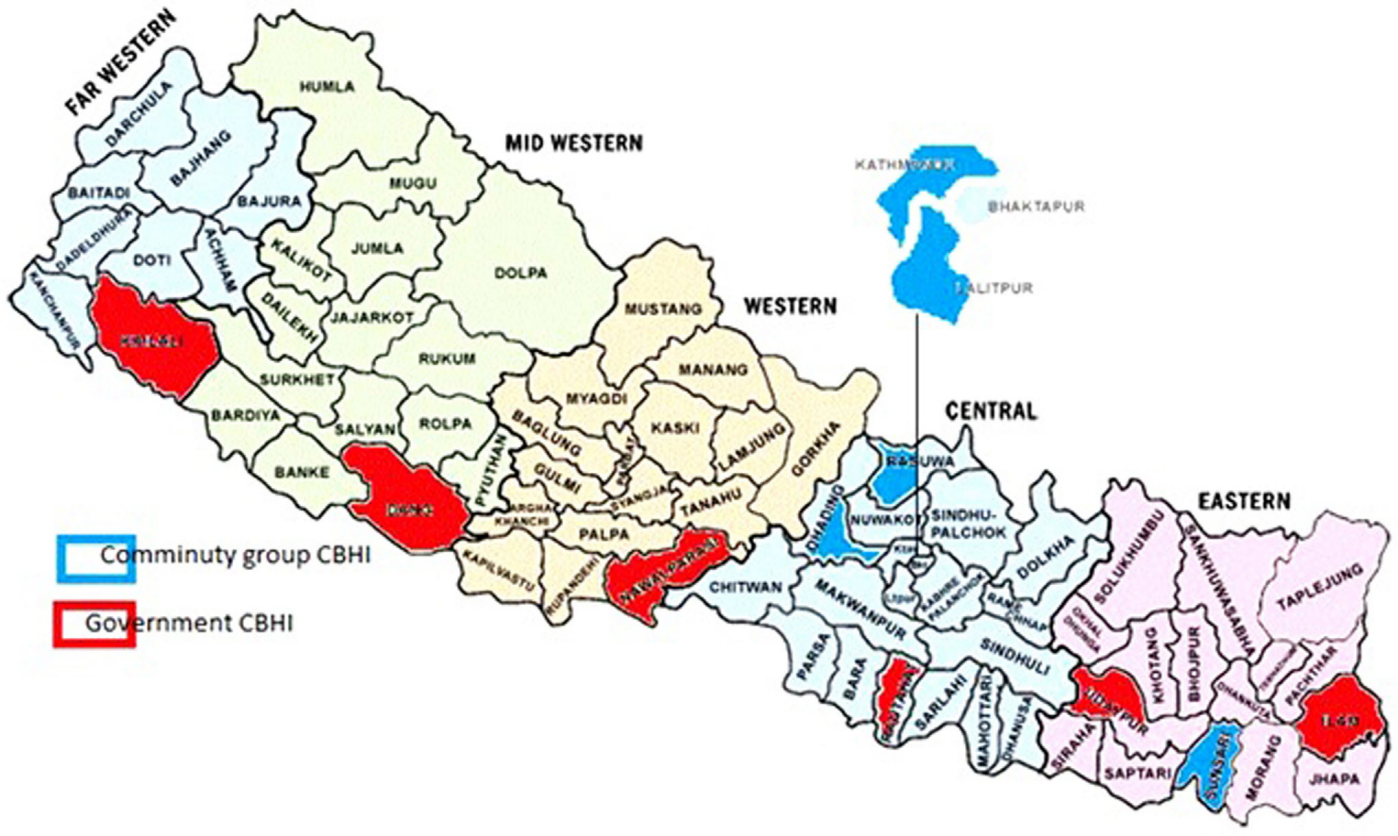
Geographical location of community-based health insurance.

The study areas were obtained from all 12 pilot organizations that had managed government health facilities and co-operative organizations (Table 2). The geographical region of community groups and government CBHI is presented in the map (Figure 2).

**Table 2:** Community-based health insurance (CBHI) operated by government and community groups.

S.N.	CBHI conducted by government (established year)	CBHI conducted by community groups (established year)
1	Lamahi Primary Health Care Centre (2006)	Madhesa health post management committee (2010)
2	Tikapur Hospital (2006)	Syaphru (2009)
3	Mangalabare Primary Health Care Centre (2004)	Rajmarga (2003)
4	Dumkauli Primary Health Care Centre (2004)	Bikalpa (2001)
5	Chandranigahapur Primary Health Care Centre (2006)	Primary Health Care and Resource Center (PHCRC), Chapagaun (1972)
6	Katari Hospital (2006)	Saubhagya (2011)

### Study Tools and Technique

Three sets of study tools were prepared for information collection.

### Review of the Records for Quantitative Data through Observation

Data were obtained from the logbooks, ledgers, enrollment registers, and meeting minutes of the health facilities and co-operative groups. From these, the characteristics and coverage levels of the enrollees were determined. The characteristics gathered included age, gender, religion, and ethnicity of enrollees. The following information was collected from the records of each group:

1. Did you have any targets for new enrollment in the past year?
  1.1. If yes, did you have a special scheme for new enrollments?
2. What was the amount of the benefit packages you provided to the enrollee?
3. From your CBHI, what population is covered?
4. What is the composition of your enrollees (minorities, disadvantaged groups, poor, etc.) in relation to social inclusion?
5. What is the utilization rate of public health services among CBHI members?
6. Do you have any official contracts with health service providers (government and private hospitals)?
  6.1. If yes, what kinds of agreements do you have (annually, biannually, etc.)?
7. Do you negotiate with health service providers within their standard price of treatment?
  7.1. If yes, how much (Rupee or percentage)?
8. Do you have your own ambulance service to use during emergencies?

### Key Informant Interview Guidelines for Qualitative Outcomes

The key informant interview guidelines were used to assess management’s experience with CBHI groups. The informants were the focal person of government health institution and the member secretary of co-ops interviewed in the presence of the chairperson in an interactive way. The key informant’s voice was recorded on a mobile phone, and the essence was presented as results. In particular, the problems and challenges were obtained from the key informant interview process.

1. When did you initiate the CBHI?
2. What problems and challenges does your CBHI face in relation to new enrollments, dropouts, reliability, and satisfaction?
3. Who supports your group, and what kind of support do they provide?
4. What suggestions do you have for the government and any supporting organizations?
5. How do you sustain the program in terms of governance and financial support?

#### Observation Checklist

The observation checklist was prepared to identify the physical infrastructure, office setting, recording and reporting status, membership cards, registration, patient records, and bank ledgers and to observe income-generating activities. Informal question–answer sessions, individual relationships, and breakfast and break time were utilized as techniques.

### Data Management

The data were divided into two parts. The numeric data were exported into Excel and analyzed using the Statistical Package for the Social Sciences (SPSS) version 22. Likewise, the qualitative information was categorized into different groups. Common information was presented in a narrative form.

### Data Analysis

In the first phase, the descriptive findings were plotted based on different categories related to enrollment, population coverage, and population composition. In the second phase, numeric data were analyzed and tested statistically by applying the Mann–Whitney *U* test as a two-group comparison. Finally, the data and qualitative information were matched and presented simultaneously.

### Research Outcome Analysis

This was a group-based study, and the main outcome was presented by comparing two group’s mean as below:


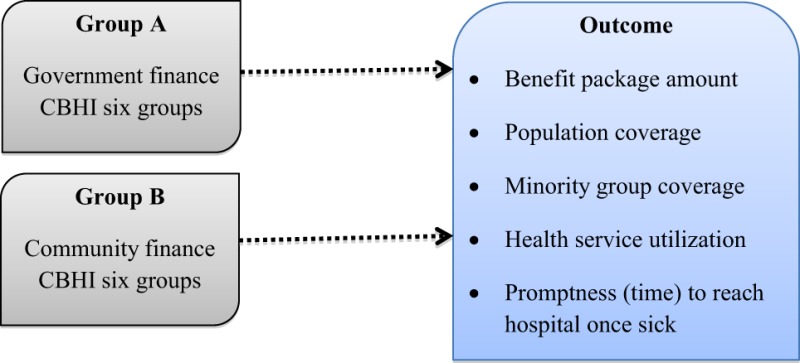


### Validity and Reliability

The results were synthesized carefully according to the data synthesis model for both qualitative and quantitative information. Numeric data were repeatedly checked by the appointed researcher and were cross-verified by another researcher. The tools were pretested in a similar community using video recordings. Necessary changes were made to the tools after the pilot study. The mixed method of descriptive study produces a high power of communicative validity, expert validity, argumentative validity, and cumulative validity compared to other methods (31).

### Ethical Consideration

Study approval was granted by the Department of Health Service (DoHS) of Teku, Kathmandu, Nepal, and a formal letter was sent to the co-operatives and government health facilities. The research was done according to international research ethical guidelines and Nepal health research council ethical guidelines (32). The respondents were clearly informed that the interviews were part of a research project and their responses might be part of a publication. Informed consent was obtained from respondents (focal person, member secretary, and chairperson of all CBHI) in accordance to the declaration of Helsinki so that it was a volunteer participation, and the information provided by respondents were confidential and it would not be used for any other purpose and respondents could withdraw their participation at anytime (33).

## Results

In this study, a comparison of the outcomes of the government and the co-operative health insurance plans in Nepal was performed. In the government CBHI, 4,364 households with 22,691 individuals were enrolled, and in the co-ops, 2,152 households with 10,106 individuals were enrolled, showing that the average family size was higher (5.2 person) in government group than that in the co-ops (4.3 person). The composition of men and women was almost equal; the adult population was dominant, and most people were of the Hindu religion and there was mixed ethnicity. Enrollment was not satisfactory (low), and population coverage was minimal. The demographic features were not much different during the piloting of the projects. The benefit package was slightly higher for the co-op group, while population coverage was better for the government group. Inclusiveness, overall management, and proxy activities were better in the co-op group (Table 3).

**Table 3:** Descriptive comparison between government and community groups community-based health insurance (CBHI).

Indicators		Government	Co-operative
**ENROLLMENT AND BENEFIT PACKAGE**			
Enrollment target		Not achieved	No fix target
Scheme		No special subsidy beyond the benefit package	Some discounts for those who want to enroll in groups and those with a poor economic status
Benefit package in Nepali rupees		Medicines, diagnostic services, hospitalization, and transportation (Rs 6,000–20,000)	Medicines, diagnostic services, hospitalization, and transportation (Rs 6,000–29,000)
Enrollment coverage (%)	New members	3.4%	2.4%
	Renewal	Negative trend up to 47%	Constant
Membership coverage in the catchment area (%)		53	26
**ENROLLMENT COMPOSITION**			
Religious minority (%)		3	36
Disadvantaged Terai (%)		2	3
Utilization of health services (%)		107 (42–162)	137 (6–230)
Receiving a discount after negotiation (%)		19	40
Scheme viability		Less viable	Average
Legal framework		No legal framework in the MoH	Legalized under co-operative law
**MANAGEMENT**			
Audit system		Rarely audited	Regularly audited
Software		Not in practice	Computer recording
Human resources for health insurance		Paramedic of hospital as focal person	Secretary of co-ops
Relationship with providers		No contract with providers	Two co-operatives have contracts with providers at district and regional hospitals
Referral service		Referred by ambulance or public vehicle to their own health center	All of them have their own ambulance
Subsidy		From the government	None
Sustainability		Depends on government funding	Have their own funds, but not sufficient
**PROXY INDICATORS**			
Income generation activities		No	Yes
			➢ Co-operative vegetable farming ➢ Poultry farming ➢ Small livestock

### Result from Qualitative Information Taken by Key Informants Interview

Qualitative information was collected from 24 participants. Among them, 12 (50%) were presidents of health centers and co-ops having intermediate education and 12 (50%) of the member secretaries were graduated from university. Eight (33.33%) participants were female and five were from disadvantage groups. Age of the respondents was 20–48 years.

A similar result was found during the key informant interviews. Both representatives reported that there was less interest in new enrollment. During the observations and conversations with key informants, the following results were found.

### Government-Operated CBHI

Although many people receive services from the government health facilities, the services that are generally provided are not promoted with insured patients to promote health insurance. There are similar types of problems and challenges for the government-operated CBHI. According to key informants’ information, people were not interested in CBHI because enrollment in CBHI did not result in their getting significant quality health service, in comparison with people not enrolled. The people who enrolled in CBHI ignored continuing membership because enrolled people who were not sick did not get any benefit from the CBHI package and felt the loss of money. On the other hand, the subsidy provided by the local government was for ultra-poor and for a limited time only. As a result, there was a high dropout rate. For the volunteer model of health insurance, availability of a pharmacy operated by hospital workers on hospital premises is a main challenge for the future of CBHI. Health workers and other personnel lacked experience in health insurance management and realized that they could not provide priority to insured patients or encourage new enrollment because the staff already had high workloads. The allocated budget was not recorded properly and used for administrative purposes (traveling and daily allowance).

### Co-Ops Operated CBHI

Community groups had different experiences. Some NGOs supported capacity building for health workers, such as training, workshops, and materials. They had a better networking and bonds with CBHI users and financial transparency, but service quality was no different than the government-operated CBHI. Involvement of all types of members in CBHI, government ignorance to guide and provide subsidy to co-ops CHBI, volunteer type of insurance models, and inability to increase the hospital service quality were the main challenges for co-ops CBHI. These co-op groups properly maintained records compared with the government health facilities, but the recording systems were not consistent. All co-ops reported that they provided awareness on sustainable health financing, and as a result, they had few dropouts. They just started to use computer-based records for each patient, expenditures, and other important decisions. They had collected more funds from their members and minimized administrative costs. Due to their strong negotiating skills, they had saved some money during service contracts with hospitals and invested this amount for other income-generating activities. There was quite a different conversation with the representative member from the co-ops.

Table 4 shows a comparison of the mean of each group variable examined in numeric data. The proportion of overall inclusiveness for the government group was significantly higher (*p* < 0.05) than that of the co-op group. However, the amount of negotiation and average response for treatment after illness/injury (initial and refer) were significantly better for the co-op group compared to that of the government health facilities.

**Table 4:** Comparison of health indicators between the two CBHI models.

Variables	Type of organization	Mean ± SD	*p*-Value
Amount of benefit package in Rupees	Government	14,333 ± 6,274	0.108
	Co-operative	45,775 ± 43,184	

Coverage population per group or health center	Government	3,781 ± 1,945	0.057
	Co-operative	1,684 ± 1,390	

Coverage of overall inclusiveness in numbers per group or health center	Government	1,930 ± 1,120	0.010
	Co-operative	417 ± 362	

Inclusiveness of religious minorities (numbers)	Government	78 ± 56	0.048
	Co-operative	547 ± 523	

Inclusiveness of disadvantaged Terai (%)	Government	64 ± 100	0.940
	Co-operative	70 ± 168	

Health service utilization rate (%)	Government	107 ± 43	0.524
	Co-operative	137 ± 102	

Proportion of discounts after negotiation (%)	Government	18 ± 10	0.003
	Co-operative	40 ± 7	

Average response for treatment after illness/injury (initial and refer) to reach hospital (min)	Government	118 ± 38	0.008
	Co-operative	38 ± 45	

The results from the qualitative and quantitative methods clearly show the effectiveness of government-operated and co-ops-operated CBHI in Nepal. Each finding complements the other.

## Discussion

The characteristics of the two models of CBHI in Nepal are clearly shown in this study. The population coverage was significantly higher in the government-conducted CBHI, but inclusiveness and institutional capacity were stronger in the co-ops. Health insurance has been in operation in Nepal for a long time on a small scale, but the Ministry of Health (MoH) has been unable to establish milestone targets for UHC. Existing government CBHI programs are not attractive to people and the co-op CBHI has poor coverage as well, but they have a positive direction. User fees, community drug programs, and free health service policies in the past have created confusion among individuals looking to enroll in CBHI (34). We found comparatively large coverage and relatively flexible premiums (in terms of payment schedules) and subsidies for the ultra-poor in the government-run CBHI. However, in this model, the local communities were unable to take ownership, and there was very low utilization of the resources. By contrast, in the co-ops, prepayment CBHI engendered trust and a feeling of ownership. A similar conclusion was drawn by Mebratie et al. (35) in a systematic review published in 2013.

In looking at our results and comparing these with other studies, we found similar trends. Enrollment is the first step in any CBHI model. Based on the large number of enrollees, the average enrollment was significantly higher in the government CBHI model, but the number of disadvantaged minorities was significantly higher in the co-op group. This finding is similar to results in a study in India that women in self-help groups found more inclusiveness among minority populations (36). In both groups, healthcare utilization increased significantly (up to two times), and this condition was also observed in the Sky Community Group in Cambodia (37); the Grameen Bank group in Bangladesh (38); the Government Amhims group in Ghana (39); Jaminan Kesehatan Aceh (JKA) scheme in Indonesia (40); and the Mutelleus Government Centre in Rwanda (41), Kerala (India) (42), and Vietnam (43). At the same time, coverage of the population in the catchment area was low in both groups. The same trend was observed in the People’s Democratic Republic of Laos (11). New enrollment and retention of current enrollees was low in both models due to uncertain financial viability, quality of care, long waiting time when seeking care, and the poor management skills of healthcare providers that was found in Ghana too (44). Yeshavani is a co-operative CBHI provider in India (45), and Urban Resident Basic Medical Insurance (URBMI) has the same function in China (46) similar to the government CBHI model in Nepal. In both cases, people from remote areas and those in the higher education class were not interested in enrollment, similar to a trend in Mali (47), and findings are similar to our results. The number of new enrollees has been decreasing in the Hanang district of Tanzania (48), as in the government CBHI in our study. Enrollment of members of the Terai disadvantaged group was significantly higher in the co-op insured group; this finding is similar to the results from the SEWA group in India (49), but the Nouna community health organization in Burkina Faso (50) and the Mutual Health Organizations in Senegal (51) have not been able to cover disadvantaged groups.

The discounts provided during service contracting with hospitals were significantly larger (*p* < 0.05) for the co-op group (by effective negotiation skills) versus the government CBHI. This is not only beneficial for the sustainability of the health insurance industry but also empowers individual and institutional capabilities.

In India, it was concluded that people’s negotiating power reduced the costs and improved the quality of service (52). In Canada, per-patient cost was 17% lower than the average price; hospitalization rates were up to 30% lower, and 21% less money was spent on prescription drugs (53) in co-operative health insurance compared to private health insurance. In China, the operating capacity of a CBHI is significantly higher in a new NCMS than it is in a government health insurance scheme (54). In addition, health service quality and profit-generating activities were launched in co-operative health insurance in Canada (55). In a Nigerian co-operative model, awareness of sustainable health financing through counseling was better, and there is a low dropout of enrollee (56). The above results from India, Canada, Nigeria, and China were similar to our findings.

The health insurance industry in Nepal has been in existence for a long time, but coverage is still low, and there are only a few successful CBHI models. The health insurance plans offered by co-operative groups are a newly growing strategic movement in health service in the country. With proper subsidies from the government and long-term contracts with advanced hospitals, health insurance offered by community groups could be more effective as an almost zero cost financing model, and health equity and quality of service in Nepal could be more accessible to people. However, as a whole, CBHI has achieved limited success in terms of community participation, self-health care, and social unity. Thus, significant support such as enough training, awareness increasing in the community, and performance-based subsidies are still necessary for both CBHI models as shown in the case of East Africa (57). There are different minority groups in Nepal and inconsistency region by region. So, overall inclusiveness of religious minorities and the disadvantaged Terai group is not in equal proportion to government-operated and co-operative groups in this study. There are some limitations in our study. There were no sufficient variables to compare co-operative and government health insurance. The results of this study are from the supply side (government health facilities and community groups), and findings may differ when the demand side (consumers/user) is examined. The interpretation of results needs treated with care generalization. In addition, some information from the key people interviewed may be more subjective, and there is a risk in generalization.

## Conclusion

Community health insurance programs have multiple and long-term impacts because they can reduce financial pressure on the government and would be responsible for maintaining health and empowering people. More importantly, it is necessary to reduce the out-of-pocket expenditures and CHEs (58) for newly designated community groups in developing countries, especially those in remote areas and for people in urban areas who cannot afford private health care. Government should provide subsidies based on the performance for both groups, and NGO should have supporting role such as training and logistic support. Ultimately, successful CBHI models could be a milestone to achieve Universal Health Coverage.

## Author Contributions

CR took overall responsibility for manuscript publication, from concept formation, study design, information collection data management, manuscript preparation, compilation of comments, and feedback to manuscript submission. C-BK monitored and suggested the framework for article preparation, review of the manuscript, cross checking of references, and adjustment of article content. MP and DS cross-checked the data and results and reviewed of the manuscript. All authors have read and approved the final manuscript.

## Conflict of Interest Statement

The authors declare that the research was conducted in the absence of any commercial or financial relationships that could be construed as a potential conflict of interest.
